# Reinvestigation of the low-temperature form of Ag_2_Se (naumannite) based on single-crystal data

**DOI:** 10.1107/S1600536811028534

**Published:** 2011-08-02

**Authors:** Jaemin Yu, Hoseop Yun

**Affiliations:** a Division of Energy Systems Research and Department of Chemistry, Ajou University, Suwon 443-749, Republic of Korea

## Abstract

The crystal structure of the low-temperature form of synthetic naumannite [disilver(I) selenide], Ag_2_Se, has been reinvestigated based on single-crystal data. In comparison with previous powder diffraction studies, anisotropic displacement parameters are additionally reported. The structure is composed of Se layers and two crystallographically independent Ag atoms. One Ag atom lies close to the Se layer and is surrounded by four Se atoms in a distorted tetra­hedral coordination, while the second Ag atom lies between the Se layers and exhibits a [3 + 1] coordination defined by three close Se atoms, forming a trigonal plane, and one remote Se atom.

## Related literature

The crystal structure of the low-temperature form of Ag_2_Se has been previously refined by using X-ray (Wiegers, 1971 ▶) and synchrotron (Billetter & Ruschewitz, 2008 ▶) powder diffraction. For the structure of the cubic high-temperature form of Ag_2_Se, see: Oliveria *et al.* (1988 ▶). For general background, see: Frueh (1958 ▶). For ionic radii, see: Shannon (1976 ▶).

## Experimental

### 

#### Crystal data

Ag_2_Se
*M* *_r_* = 294.7
Orthorhombic, 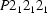

*a* = 4.3359 (8) Å
*b* = 7.070 (1) Å
*c* = 7.774 (1) Å
*V* = 238.34 (7) Å^3^
*Z* = 4
Mo *K*α radiation
μ = 31.27 mm^−1^
*T* = 290 K
0.30 × 0.04 × 0.02 mm

#### Data collection

Rigaku R-AXIS RAPID diffractometer
Absorption correction: multi-scan (*NUMABS*; Higashi, 2000 ▶) *T*_min_ = 0.053, *T*_max_ = 0.278
1981 measured reflections
464 independent reflections
447 reflections with *I* > 2σ(*I*)
*R*_int_ = 0.057

#### Refinement

*R*[*F* ^2^ > 2σ(*F* ^2^)] = 0.031
*wR*(*F* ^2^) = 0.080
*S* = 1.14
464 reflections
29 parameters
Δρ_max_ = 1.19 e Å^−3^
Δρ_min_ = −1.07 e Å^−3^
Absolute structure: Flack (1983 ▶), 167 Friedel pairs
Flack parameter: 0.34 (4)

### 

Data collection: *RAPID-AUTO* (Rigaku, 2006 ▶); cell refinement: *RAPID-AUTO*; data reduction: *RAPID-AUTO*; program(s) used to solve structure: *SHELXS97* (Sheldrick, 2008 ▶); program(s) used to refine structure: *SHELXL97* (Sheldrick, 2008 ▶); molecular graphics: locally modified version of *ORTEP* (Johnson, 1965 ▶); software used to prepare material for publication: *WinGX* (Farrugia, 1999 ▶).

## Supplementary Material

Crystal structure: contains datablock(s) global, I. DOI: 10.1107/S1600536811028534/wm2506sup1.cif

Structure factors: contains datablock(s) I. DOI: 10.1107/S1600536811028534/wm2506Isup2.hkl

Additional supplementary materials:  crystallographic information; 3D view; checkCIF report

## Figures and Tables

**Table 1:** Selected bond lengths (Å)

Ag1—Se^i^	2.6800 (14)
Ag1—Se^ii^	2.7058 (16)
Ag1—Se^iii^	2.8282 (14)
Ag1—Se^iv^	2.9076 (16)
Ag2—Se^iii^	2.6538 (14)
Ag2—Se	2.7560 (15)
Ag2—Se^v^	2.8036 (16)
Ag2—Se^vi^	3.2112 (16)

Symmetry codes: (i) 
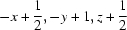
; (ii) 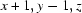; (iii) 
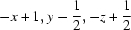
; (iv) 
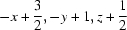
; (v) 
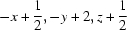
; (vi) 
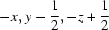
.

**Fig. 1.**
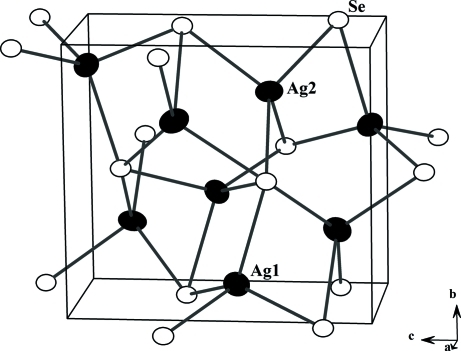
View of Ag2Se (50% probability displacement ellipsoids)

## supplementary crystallographic information

### Comment

Structural studies of the low-temperature form (transition point is 406 K) of the mineral naumannite, Ag_2_Se, based on powder diffraction data have been reported previously by Wiegers (1971; X-ray data) and Billetter & Ruschewitz (2008; synchrotron data). In case of the related phase Ag_2_S, single crystals of the high-temperature form (space group *Im*3*m*) are known to convert to polycrystalline powder of the low-temperature form on cooling (Frueh, 1958), and the same conversion has been assumed for the Se analogue (Wiegers, 1971). Consequently, structure determinations of the low-temperature form of Ag_2_Se have been carried out only by using powder diffraction methods. In an attempt to prepare new mixed-metal selenides using AgCl as a flux, we were able to isolate single crystals of the low-temperature form of Ag_2_Se and report here the results of the structure analysis. In comparison with the previous powder diffraction studies, anisotropic displacement parameters are additionally reported.

The general structural features of AgSe_2_ are the same as reported previously (Wiegers, 1971; Billetter & Ruschewitz, 2008). The structure of the low-temperature form of Ag_2_Se is closely related to the cubic high-temperature phase, where the Se atoms form a body-centered cubic packing, while the silver atoms are statistically distributed over interstitial sites (Oliveria *et al.*, 1988). As a result, layers composed of Se atoms perpendicular to the *b* axis are retained in the low-temperature structure (Fig. 1). There are two crystallographically independent Ag atoms. Ag1 atoms lie close to this layer and are surrounded by four Se atoms in a distorted tetrahedral fashion (Se—Ag1—Se, 91.55 (3)–136.30 (5) °). The Ag1—Se distances range from 2.6800 (14) Å to 2.9076 (16) Å. Ag2 atoms are located between the layers and the coordination can be described as [3+1]. Three Se atoms built up a triangle that is bound to the Ag2 atom (Se—Ag2—Se, 94.00 (3)–141.46 (5) °), the coordination of which is augmented by a forth Se atom at a considerably longer distance of 3.2112 (16) Å. The observed Ag—Se distances are in agreement with the sum of the ionic radii of each element (Shannon, 1976) except for the very long Ag—Se bond. The distances and angles as calculated from single-crystal diffraction data differ slightly from those calculated previously from powder diffraction data. For example, the reported Ag1—Se distances are 2.62, 2.71, 2.79, 2.86 Å (Wiegers, 1971) and 2.658 (4), 2.668 (5), 2.861 (5), 2.937 (5) Å (Billetter & Ruschewitz, 2008) Å, and the Ag2—Se distances are 2.72, 2.74, 2.81, 3.28 Å (Wiegers, 1971) and 2.686 (5), 2.764 (5), 2.797 (4), 3.182 (5) Å (Billetter & Ruschewitz, 2008), respectively, with lattice parameters of  *a* = 4.333 (Wiegers, 1971); 4.3373 (2) Å (Billetter & Ruschewitz, 2008), *b* = 7.062; 7.0702 (3) Å; *c* = 7.764; 7.7730 (4) Å.

### Experimental

Single crystals of the low-temperature form of Ag_2_Se were isolated during attemts to prepare new mixed-metal phases of Hf/Zr selenides. A combination of the pure elements, Zr powder, Hf powder, Se powder were mixed in a fused silica tube in a molar ratio of Zr: Hf: Se = 1:1:3 with AgCl. The mass ratio of the reactants and the halide flux was 1:2. The tube was evacuated to 0.133 Pa, sealed and heated gradually (20 K/h) to 600 K, where it was kept for 72 h. The tube was cooled to 200 K at 3 K/h and then was quenched to room temperature. The excess halide flux was removed with distilled water and black needle shaped crystals were obtained. The crystals are stable in air and water. A qualitative XRF analysis of the crystals showed only the presence of Ag and Se.

### Refinement

Refinement with TWIN and BASF instruction for the final positional parameters gave a value of 0.34 (4) for the Flack parameter (Flack, 1983). The highest peak and the deepest hole in the final Fourier map are located 1.71 Å and 0.99 Å, respectively, from atom Ag1.

### Crystal data

**Table:**

Ag_2_Se	*F*(000) = 512
*M**_r_* = 294.7	*D*_x_ = 8.213 Mg m^−^^3^
Orthorhombic, *P*2_1_2_1_2_1_	Mo *K*α radiation, λ = 0.71073 Å
Hall symbol: P 2ac 2ab	Cell parameters from 1738 reflections
*a* = 4.3359 (8) Å	θ = 3.4–27.5°
*b* = 7.070 (1) Å	µ = 31.27 mm^−^^1^
*c* = 7.774 (1) Å	*T* = 290 K
*V* = 238.34 (7) Å^3^	Needle, black
*Z* = 4	0.30 × 0.04 × 0.02 mm

### Data collection

**Table:**

Rigaku R-AXIS RAPID diffractometer	447 reflections with *I* > 2σ(*I*)
ω scans	*R*_int_ = 0.057
Absorption correction: multi-scan (*NUMABS*; Higashi, 2000)	θ_max_ = 26.0°, θ_min_ = 3.9°
*T*_min_ = 0.053, *T*_max_ = 0.278	*h* = −5→5
1981 measured reflections	*k* = −8→8
464 independent reflections	*l* = −9→9

### Refinement

**Table:**

Refinement on *F*^2^	0 restraints
Least-squares matrix: full	*w* = 1/[σ^2^(*F*_o_^2^) + (0.0329*P*)^2^ + 1.0072*P*] where *P* = (*F*_o_^2^ + 2*F*_c_^2^)/3
*R*[*F*^2^ > 2σ(*F*^2^)] = 0.031	(Δ/σ)_max_ < 0.001
*wR*(*F*^2^) = 0.080	Δρ_max_ = 1.19 e Å^−^^3^
*S* = 1.14	Δρ_min_ = −1.07 e Å^−^^3^
464 reflections	Absolute structure: Flack (1983), 167 Friedel pairs
29 parameters	Flack parameter: 0.34 (4)

### Special details

**Table:**

Geometry. All e.s.d.'s (except the e.s.d. in the dihedral angle between two l.s. planes) are estimated using the full covariance matrix. The cell e.s.d.'s are taken into account individually in the estimation of e.s.d.'s in distances, angles and torsion angles; correlations between e.s.d.'s in cell parameters are only used when they are defined by crystal symmetry. An approximate (isotropic) treatment of cell e.s.d.'s is used for estimating e.s.d.'s involving l.s. planes.

### Fractional atomic coordinates and isotropic or equivalent isotropic displacement parameters (Å^2^)

**Table:**

	*x*	*y*	*z*	*U*_iso_*/*U*_eq_	
Ag1	0.8537 (2)	0.11503 (14)	0.45100 (14)	0.0398 (3)	
Ag2	0.4745 (3)	0.77441 (14)	0.36152 (14)	0.0447 (4)	
Se	0.1124 (2)	0.99787 (14)	0.15274 (14)	0.0242 (3)	

### Atomic displacement parameters (Å^2^)

**Table:**

	*U*^11^	*U*^22^	*U*^33^	*U*^12^	*U*^13^	*U*^23^
Ag1	0.0381 (6)	0.0378 (6)	0.0436 (6)	−0.0010 (5)	0.0080 (5)	0.0060 (4)
Ag2	0.0462 (7)	0.0338 (6)	0.0540 (7)	0.0131 (4)	−0.0100 (5)	−0.0057 (5)
Se	0.0249 (5)	0.0190 (6)	0.0286 (6)	0.0001 (4)	−0.0011 (4)	−0.0006 (4)

### Geometric parameters (Å, °)

**Table:**

Ag1—Se^i^	2.6800 (14)	Ag2—Se^ix^	3.2112 (16)
Ag1—Se^ii^	2.7058 (16)	Ag2—Ag1^x^	2.9979 (16)
Ag1—Se^iii^	2.8282 (14)	Ag2—Ag1^xi^	3.0330 (14)
Ag1—Se^iv^	2.9076 (16)	Ag2—Ag2^xii^	3.0749 (16)
Ag1—Ag1^v^	2.9872 (15)	Ag2—Ag2^xiii^	3.0749 (16)
Ag1—Ag1^vi^	2.9872 (15)	Ag2—Ag1^vi^	3.1591 (16)
Ag1—Ag2^vii^	2.9979 (15)	Ag2—Ag1^xiv^	3.3692 (17)
Ag1—Ag2^iii^	3.0330 (14)	Se—Ag2^xi^	2.6538 (14)
Ag1—Ag2^v^	3.1591 (16)	Se—Ag1^xv^	2.6800 (14)
Ag1—Ag2^iv^	3.3692 (17)	Se—Ag1^xvi^	2.7058 (16)
Ag2—Se^iii^	2.6538 (14)	Se—Ag2^xvii^	2.8036 (16)
Ag2—Se	2.7560 (15)	Se—Ag1^xi^	2.8282 (14)
Ag2—Se^viii^	2.8036 (16)	Se—Ag1^xiv^	2.9076 (16)
			
Se^i^—Ag1—Se^ii^	136.30 (5)	Se^viii^—Ag2—Ag1^x^	54.90 (4)
Se^i^—Ag1—Se^iii^	119.41 (5)	Se^iii^—Ag2—Ag1^xi^	90.54 (4)
Se^ii^—Ag1—Se^iii^	91.55 (3)	Se—Ag2—Ag1^xi^	58.26 (4)
Se^i^—Ag1—Se^iv^	101.71 (5)	Se^viii^—Ag2—Ag1^xi^	142.86 (5)
Se^ii^—Ag1—Se^iv^	92.76 (3)	Ag1^x^—Ag2—Ag1^xi^	137.88 (5)
Se^iii^—Ag1—Se^iv^	112.04 (5)	Se^iii^—Ag2—Ag2^xii^	58.04 (3)
Se^iii^—Ag2—Se^ix^	94.87 (4)	Se—Ag2—Ag2^xii^	151.45 (6)
Se—Ag2—Se^ix^	82.95 (3)	Se^viii^—Ag2—Ag2^xii^	66.03 (5)
Se^viii^—Ag2—Se^ix^	105.72 (5)	Ag1^x^—Ag2—Ag2^xii^	62.68 (4)
Se^i^—Ag1—Ag1^v^	126.05 (6)	Ag1^xi^—Ag2—Ag2^xii^	148.22 (5)
Se^ii^—Ag1—Ag1^v^	96.50 (4)	Se^iii^—Ag2—Ag2^xiii^	114.98 (6)
Se^iii^—Ag1—Ag1^v^	54.80 (4)	Se—Ag2—Ag2^xiii^	94.31 (4)
Se^iv^—Ag1—Ag1^v^	57.32 (3)	Se^viii^—Ag2—Ag2^xiii^	53.43 (4)
Se^i^—Ag1—Ag1^vi^	59.58 (3)	Ag1^x^—Ag2—Ag2^xiii^	108.32 (6)
Se^ii^—Ag1—Ag1^vi^	135.65 (6)	Ag1^xi^—Ag2—Ag2^xiii^	100.86 (4)
Se^iii^—Ag1—Ag1^vi^	59.92 (5)	Ag2^xii^—Ag2—Ag2^xiii^	89.67 (6)
Se^iv^—Ag1—Ag1^vi^	127.99 (6)	Se^iii^—Ag2—Ag1^vi^	59.27 (4)
Ag1^v^—Ag1—Ag1^vi^	93.06 (6)	Se—Ag2—Ag1^vi^	132.61 (5)
Se^i^—Ag1—Ag2^vii^	58.86 (4)	Se^viii^—Ag2—Ag1^vi^	96.07 (5)
Se^ii^—Ag1—Ag2^vii^	77.44 (4)	Ag1^x^—Ag2—Ag1^vi^	133.16 (4)
Se^iii^—Ag1—Ag2^vii^	136.98 (5)	Ag1^xi^—Ag2—Ag1^vi^	88.17 (3)
Se^iv^—Ag1—Ag2^vii^	109.95 (4)	Ag2^xii^—Ag2—Ag1^vi^	72.30 (4)
Ag1^v^—Ag1—Ag2^vii^	166.05 (5)	Ag2^xiii^—Ag2—Ag1^vi^	57.47 (4)
Ag1^vi^—Ag1—Ag2^vii^	100.04 (3)	Se^iii^—Ag2—Ag1^xiv^	88.98 (4)
Se^i^—Ag1—Ag2^iii^	103.04 (4)	Se—Ag2—Ag1^xiv^	55.60 (4)
Se^ii^—Ag1—Ag2^iii^	67.77 (4)	Se^viii^—Ag2—Ag1^xiv^	131.41 (5)
Se^iii^—Ag1—Ag2^iii^	55.96 (3)	Ag1^x^—Ag2—Ag1^xiv^	84.96 (4)
Se^iv^—Ag1—Ag2^iii^	155.18 (5)	Ag1^xi^—Ag2—Ag1^xiv^	55.32 (3)
Ag1^v^—Ag1—Ag2^iii^	107.91 (5)	Ag2^xii^—Ag2—Ag1^xiv^	122.24 (4)
Ag1^vi^—Ag1—Ag2^iii^	68.06 (4)	Ag2^xiii^—Ag2—Ag1^xiv^	147.69 (4)
Ag2^vii^—Ag1—Ag2^iii^	81.67 (3)	Ag1^vi^—Ag2—Ag1^xiv^	132.52 (4)
Se^i^—Ag1—Ag2^v^	66.13 (4)	Ag2^xi^—Se—Ag1^xv^	134.75 (5)
Se^ii^—Ag1—Ag2^v^	93.43 (4)	Ag2^xi^—Se—Ag1^xvi^	95.17 (4)
Se^iii^—Ag1—Ag2^v^	163.17 (5)	Ag1^xv^—Se—Ag1^xvi^	106.26 (4)
Se^iv^—Ag1—Ag2^v^	51.68 (3)	Ag2^xi^—Se—Ag2	93.59 (4)
Ag1^v^—Ag1—Ag2^v^	108.60 (5)	Ag1^xv^—Se—Ag2	127.12 (5)
Ag1^vi^—Ag1—Ag2^v^	124.02 (5)	Ag1^xvi^—Se—Ag2	84.66 (5)
Ag2^vii^—Ag1—Ag2^v^	59.85 (3)	Ag2^xi^—Se—Ag2^xvii^	68.53 (4)
Ag2^iii^—Ag1—Ag2^v^	140.46 (4)	Ag1^xv^—Se—Ag2^xvii^	66.24 (4)
Se^i^—Ag1—Ag2^iv^	71.43 (4)	Ag1^xvi^—Se—Ag2^xvii^	117.42 (5)
Se^ii^—Ag1—Ag2^iv^	142.30 (5)	Ag2—Se—Ag2^xvii^	151.82 (5)
Se^iii^—Ag1—Ag2^iv^	92.10 (4)	Ag2^xi^—Se—Ag1^xi^	131.16 (5)
Se^iv^—Ag1—Ag2^iv^	51.45 (3)	Ag1^xv^—Se—Ag1^xi^	65.62 (4)
Ag1^v^—Ag1—Ag2^iv^	56.62 (4)	Ag1^xvi^—Se—Ag1^xi^	123.98 (5)
Ag1^vi^—Ag1—Ag2^iv^	76.74 (5)	Ag2—Se—Ag1^xi^	65.78 (4)
Ag2^vii^—Ag1—Ag2^iv^	121.83 (4)	Ag2^xvii^—Se—Ag1^xi^	109.05 (5)
Ag2^iii^—Ag1—Ag2^iv^	140.93 (4)	Ag2^xi^—Se—Ag1^xiv^	69.05 (4)
Ag2^v^—Ag1—Ag2^iv^	74.25 (3)	Ag1^xv^—Se—Ag1^xiv^	101.71 (5)
Se^iii^—Ag2—Se	141.46 (5)	Ag1^xvi^—Se—Ag1^xiv^	151.20 (5)
Se^iii^—Ag2—Se^viii^	123.20 (5)	Ag2—Se—Ag1^xiv^	72.95 (4)
Se—Ag2—Se^viii^	94.00 (3)	Ag2^xvii^—Se—Ag1^xiv^	80.16 (4)
Se^iii^—Ag2—Ag1^x^	103.38 (5)	Ag1^xi^—Se—Ag1^xiv^	62.75 (4)
Se—Ag2—Ag1^x^	89.34 (4)		

Symmetry codes: (i) −*x*+1/2, −*y*+1, *z*+1/2; (ii) *x*+1, *y*−1, *z*; (iii) −*x*+1, *y*−1/2, −*z*+1/2; (iv) −*x*+3/2, −*y*+1, *z*+1/2; (v) *x*+1/2, −*y*+1/2, −*z*+1; (vi) *x*−1/2, −*y*+1/2, −*z*+1; (vii) *x*, *y*−1, *z*; (viii) −*x*+1/2, −*y*+2, *z*+1/2; (ix) −*x*, *y*−1/2, −*z*+1/2; (x) *x*, *y*+1, *z*; (xi) −*x*+1, *y*+1/2, −*z*+1/2; (xii) *x*+1/2, −*y*+3/2, −*z*+1; (xiii) *x*−1/2, −*y*+3/2, −*z*+1; (xiv) −*x*+3/2, −*y*+1, *z*−1/2; (xv) −*x*+1/2, −*y*+1, *z*−1/2; (xvi) *x*−1, *y*+1, *z*; (xvii) −*x*+1/2, −*y*+2, *z*−1/2.
